# Cortisol in Saliva, Urine, Feces, and Hair as Non- and Minimally Invasive Measures of Adrenocortical Activity in Intensively Housed Pigs

**DOI:** 10.3390/ani16203175

**Published:** 2026-10-10

**Authors:** Levente Kovács, Márton Orbán, Balázs Gregosits, Márta Erdélyi, Miklós Mézes

**Affiliations:** 1 Department of Animal Husbandry and Animal Welfare, Institute of Animal Sciences, Hungarian University of Agriculture and Life Sciences, Páter Károly utca 1, H-2100 Godollo, Hungary; 2 Vitafort First Hungarian Feed Production and Distribution Zrt., Szabadság u. 3, H-2370 Dabas, Hungary; orban.m@vitafortasia.com (M.O.);; 3 Department of Feed Safety, Institute of Animal Physiology and Nutrition, Hungarian University of Agriculture and Life Sciences, Páter Károly utca 1, H-2100 Godollo, Hungary; ballane.erdelyi.marta@uni-mate.hu (M.E.); mezes.miklos@uni-mate.hu (M.M.)

**Keywords:** salivary cortisol, urinary cortisol, fecal glucocorticoid metabolites, hair cortisol, sow, non- and minimally invasive sampling, stress, welfare assessment, hypothalamic–pituitary–adrenal axis

## Abstract

Blood cortisol measurements may be confounded by sampling-induced handling and restraint, motivating the use of alternative matrices to assess adrenocortical activity in pigs. This narrative review examines the physiological time windows, collection requirements, analytical validation, and interpretive limitations of saliva, urine, feces, and hair. Saliva captures rapid responses but remains sensitive to collection conditions; urine integrates secretion over bladder filling and requires dilution correction; fecal glucocorticoid metabolites reflect delayed, integrated excretion and require a species-appropriate assay. Hair offers a potentially longer integration window, but controlled validation in pigs remains inconsistent, and clipping generally requires brief restraint. Antibody specificity, sample processing, and normalization constrain comparisons between studies. Published concentration benchmarks should not be interpreted as validated, production-stage-specific reference intervals. The practical matrix-selection framework presented here rests primarily on cross-study inference rather than direct comparisons of all four matrices under a common challenge. Cortisol indicates adrenocortical activation without establishing its cause or emotional valence. Interpretation therefore requires complementary behavioral, clinical, and production measures. A minimum reporting standard and priorities for paired-matrix validation, standardized sampling, and category-specific reference intervals are proposed. These matrices support comparative monitoring; none should be treated as a standalone diagnostic test of compromised welfare.

## Simple Summary

Pigs raised indoors encounter a long list of challenges they cannot escape: regrouping into unfamiliar groups, restricted feeding, barren floors, high stocking densities, heat, and the routine handling that accompanies breeding and transport. All of these activate the stress axis and release cortisol. Measuring cortisol in blood is problematic, because catching and restraining a pig raises cortisol within minutes and the measurement then partly reflects the sampling itself. Four alternatives avoid or nearly avoid handling: saliva, urine, feces, and hair. They are not equivalent. Saliva contains the hormone in its biologically active form and responds within minutes; urine sums up the period since the animal last urinated; feces reflects the period of gut passage but contains only the breakdown products left after the liver and gut bacteria have transformed the hormone; hair may reflect a longer period of weeks to months, although this has not been established in pigs, and requires the animal to be caught briefly and has given inconsistent results in controlled tests. This review outlines what pigs are exposed to in intensive systems, distinguishes collection methods that require no contact with the animal from those that require brief restraint, explains how cortisol reaches each of the four matrices, and appraises what has actually been demonstrated for each of them in pigs. We conclude that the matrix should be chosen to match the time scale of the question, and that all four are best used to compare groups and identify where stress is elevated rather than as stand-alone diagnostic tests.

## 1. Introduction and Scope

Cortisol is a steroid hormone released into the circulation in response to stressors and to falling blood glucose in most mammals [1]. Its secretion is not constant: concentrations are highest late at night and in the early morning and fall through the afternoon and early night, a rhythm that must be accounted for whenever a single sample is used to characterize an animal [2,3]. Around this rhythm, the hypothalamic–pituitary–adrenal (HPA) axis superimposes the response to challenge, and glucocorticoids act permissively, suppressively, stimulatorily, and preparatively depending on context [4]. The concept of a stereotyped response to diverse noxious agents dates to Selye [5,6] and has been progressively refined since [7,8].

The practical problem in pig production is that the animals concerned cannot avoid the challenges they meet. Pigs housed indoors are regrouped into unfamiliar social groups, fed restricted rations on barren floors, kept at high stocking densities, and handled repeatedly for breeding, vaccination, and transport. Each of these has been shown to raise glucocorticoid output [9,10,11,12], and their cumulative effect is what welfare assessment attempts to quantify [13,14]. Reviews of the field have repeatedly called for indicators that can be applied at scale without adding to the load they are meant to measure [15,16,17]. A systematic review of transport and slaughter has shown in addition that much of the load imposed at these stages arises from the procedures and human–animal interactions themselves rather than from the journey as such [18]. Section 1.1 sets out this stressor profile in detail, because the interpretation of any cortisol value depends on knowing what the animal was exposed to.

Measuring cortisol in blood introduces a potential methodological confound: restraint associated with sampling can itself elicit a cortisol response within minutes; therefore, the measured concentration may reflect both the animal’s preceding state and its response to handling [7,19]. Cook and colleagues reported increases in both serum and salivary cortisol following nose-snare restraint, with correlated concentrations in the two matrices [19]. Saliva therefore offers a less invasive sampling matrix, but it is not insensitive to restraint-induced stress. The implication is that cortisol measurements in either matrix must be interpreted in relation to the timing and conditions of collection, with handling minimized and standardized when the aim is to characterize the animal’s pre-existing state.

Three matrices can reduce this sampling-related disturbance, and the differences between them are physiological rather than merely practical. Saliva contains the free cortisol fraction, delivered by passive diffusion; it constitutes 8.6–13.3% of the serum concentration in pigs and is essentially an ultrafiltrate of the unbound fraction, whereas most commercial immunoassays applied to blood measure bound plus free hormone together [19]. Urine accumulates cortisol and its hepatic conjugates over the period of bladder filling [20,21,22]. Feces contain neither cortisol nor its hepatic conjugates but the products of subsequent bacterial transformation, integrated over the period of gut passage [23,24,25]. A fourth matrix, hair, is considered a potential indicator of longer-term exposure and is treated here alongside the other three, although it is minimally rather than fully non-invasive because the animal must be caught for clipping. Its performance in pigs has been inconsistent: repeated adrenocorticotropic hormone (ACTH) administration failed to raise it [26], although a sustained housing challenge did [27], wash protocol and external contamination alter the measured concentration [28], and season, body site, sex, and breeding system all contribute to variation [29,30,31,32,33].

Reference values are a persistent weakness across all of them. Cortisol is not routinely measured in farm animal clinical practice, and published reference intervals are correspondingly scarce; the serum concentration benchmark most often cited for pigs, irrespective of category, is 27.5–31.8 ng/mL [34]. When commercial herds are surveyed against this benchmark, most values fall outside it: in a six-farm study, only 4 of 29 serum and 5 of 30 oral fluid samples were within the cited benchmark range [35]. Whether this indicates widespread elevated load or simply an unsuitable benchmark cannot be resolved from the data, and the same ambiguity affects urine and feces.

Age is a further systematic determinant. Cortisol concentrations decline modestly with age in pigs [3], as they do in rodents [36] and humans [37,38], and weaners consistently show higher concentrations than older animals on the same farm [39]. Under sustained stress, however, both the absolute values and the age-related trajectory can be substantially altered [20], which limits the usefulness of age-unadjusted comparisons. Genetic background contributes as well, since HPA reactivity is heritable and has been proposed as a selection criterion for robustness [40].

### 1.1. Stressors in Intensive Pig Production

Any cortisol value is interpretable only against the exposure that produced it. Intensive systems present a stressor profile that is unusual in two respects: the challenges are chronic rather than episodic, and the animal has little scope to avoid them behaviorally. Lagoda and colleagues cataloged the risk factors for chronic stress in group-housed pregnant sows and grouped them into physical and social categories, noting that several act simultaneously and interact [9]. The following summary follows that structure.

Mixing unfamiliar animals is the single most consistently documented stressor. Regrouping raises glucocorticoid output and aggressive interaction frequency together, and the association between the two is positive [10,41]. The effect depends on group structure: dynamic groups, in which animals are added and removed repeatedly, impose recurrent renegotiation of social rank, whereas stable groups permit a hierarchy to settle [9,42]. Dominance status and familiarity both modulate the endocrine response to social confrontation in growing pigs [43], and social rank in pregnant sows affects body weight gain, behavior, and subsequent offspring performance [44]. Social and thermal challenge are moreover not additive in a simple way: when the two were applied together, their combined effect on immune measures differed from the effect of either alone [45].

Space allowance in the days immediately after grouping is a modifiable determinant of how severe this period becomes: both group-level and individual behavior in early gestation differ according to the area available in the first days after mixing [46]. Management can also moderate the response, since the presence of a boar at mixing altered agonistic behavior, shoulder scratching, and the stress response of newly bred sows [47]. Enrichment provision interacts with social status, with access to and use of enrichment differing between dominant and subordinate sows under programmed sow feeding [48].

Gestating sows are typically fed restricted rations to control body condition, which leaves them in a state of unresolved feeding motivation for much of the day. Feed deprivation produces a graded glucocorticoid response, detectable after 12 h and substantially larger after 24 h [11], and the inability to forage on barren floors sustains the underlying motivation even when nutritional requirements are met [9,11]. Competitive feeding systems add a social dimension to the same problem [42,48]. The feeding system itself leaves a measurable endocrine signature at herd level: salivary cortisol during gestation differed between herds according to whether sows were fed by programmed sow feeders, floor feeding, or feeding stalls, which makes the feeding system a legitimate object of welfare monitoring rather than merely a husbandry detail [49].

Dietary strategies that increase satiety or prolong feeding time act directly on this stressor. High-fiber diets combined with more frequent feeding altered behavior, reproductive performance, and nutrient digestibility in gestating sows [50], and forage-based approaches have been examined in the same spirit: inclusion of Italian ryegrass silage changed feed intake and behavior in pregnant sows [51], and crimson clover silage altered nutrient intake, behavior, and stress condition [52]. Supplementation can also act on the response to social challenge rather than on hunger: a magnesium-rich marine extract fed to growing pigs altered behavior, salivary cortisol, and skin lesion scores, linking a dietary intervention to an agonistic outcome [53]. These interventions target the motivational cause rather than the endocrine consequence, which is one reason their effects on behavior are often clearer than their effects on cortisol.

Floor type, pen design, and the absence of rooting substrate all contribute to chronic load [9]. Straw bedding alters physiological responses to stressors in growing pigs, and environmental enrichment modifies both behavioral reactivity and the circadian cortisol profile [2,20]. The gestational environment leaves traces well beyond behavior, influencing maternal physiology and milk secretions in ways relevant to neonatal survival [54], and the farrowing environment itself shapes the physiology of the sow through late pregnancy and early lactation [55,56]. Lighting is an underappreciated component: light emitted by piglet heat lamps altered sow behavior and piglet salivary melatonin concentrations [57]. Locomotor disorders deserve separate mention, since lameness both results from and contributes to chronic stress, and early detection in gilts is associated with chronic stress markers and reproductive outcome [58]. Restriction of movement is a stressor in its own right: confinement of primiparous sows during lactation altered both HPA function and behavior, and the two responses did not run in parallel [59]. Clinical conditions of this kind can be scored directly; a composite score for pain, distress, and discomfort in pigs with lameness or prolapse correlated with salivary biomarkers and with disease severity, which offers a way of anchoring an endocrine measurement to an observable clinical state [60].

Thermal load is the environmental stressor with the clearest endocrine signature. In periparturient sows, the thermal environment altered HPA hormones, oxytocin, and behavioral activity [61], and controlled heat stress in late gestation affected thermoregulation, productive performance, and metabolite profiles in primiparous animals [62]. Gestational heat stress also has consequences that persist into the next generation, with reproductive efficiency and thermoregulation differing between replacement gilts divergently selected for thermotolerance [63]. In growing pigs reared at high ambient temperature, hair cortisone and the cortisone-to-cortisol ratio both rose in uncooled rooms, whereas hair cortisol itself did not change detectably [30]. The consequences extend to the product as well as to the animal, since chronic prenatal and postnatal heat stress alters pork quality traits and the associated muscle metabolic profile [64], and the scale of the problem together with the available mitigation strategies has recently been reviewed [65]. Housing system as a whole leaves a detectable signature, with intensive systems generally associated with higher basal concentrations than extensive ones [39,66].

Because heat stress is expected to become more frequent, mitigation through the diet has attracted attention. Dietary electrolyte balance influenced performance, inflammation, and gut integrity in lactating sows under heat stress [67]; adjusting dietary mineral level of the feed altered the response of lactating sows [68]; and supplementation with a host-derived *Limosilactobacillus reuteri* improved piglet growth and gut integrity in heat-stressed sows [69]. These studies measure endpoints downstream of the HPA axis rather than cortisol itself, which limits direct comparison but broadens the picture of what thermal load does.

Routine husbandry generates acute peaks superimposed on the chronic background. Handling quality at slaughter is discriminated by salivary analytes [70], road transport raises fecal glucocorticoid metabolites with a peak 12 to 24 h after the journey [25], and reproductive interventions such as transvaginal ovum pick-up impose a measurable welfare cost [71]. A controlled comparison of acute stressors in pigs showed that behavioral, endocrine, immune, and performance measures do not respond in concert; therefore, the choice of endpoint determines which stressors appear severe [72]. Transport is the best-characterized of these events: summer transport of cull sows altered behavioral and physiological stress biomarkers, and the response differed between animals selected for heat stress tolerance and for sensitivity [73], while a systematic review of transport and slaughter identified handling procedures and human–animal interaction as the dominant modifiable determinants [18]. Conditioning can attenuate the response, as a kennel conditioning protocol reduced transport-induced changes in heart rate variability and salivary cortisol in minipigs [74]. Because these events are brief and the matrices differ in temporal resolution, the same event may be conspicuous in saliva and invisible in feces.

Weaning is the most abrupt transition in the production cycle and combines maternal separation, mixing, and a dietary change. It elicits a three- to fourfold glucocorticoid rise within 24 to 48 h [12], and recent work shows that the endocrine disturbance is not confined to the adrenal axis: weaning altered both the hypothalamic–pituitary–thyroid (HPT) and HPA axes in pigs; therefore, cortisol captures only part of the response [75]. Early-life experience modifies how animals meet these later challenges, since manipulating the environment and handling of young pigs altered growth trajectories, immune and stress responses, clinical indicators, and intestinal health [76], and preweaning housing left detectable behavioral and physiological traces through the suckling and fattening periods [77].

Not every intervention is aversive. Playback of artificial piglet suckling sounds influenced piglet behavior and performance and altered adrenal responses in sows [78], illustrating that the acoustic environment is a manipulable variable rather than a fixed background. Nurse sow strategies, by contrast, extend the lactational burden of selected animals and have consequences for several measures of sow welfare [79].

Chronic stress in the pregnant sow is not confined to her own welfare. Prenatal social stress alters neuroendocrine activity and behavior in the offspring [80], and the resilience of the piglet is mediated in part by the gestational environment of the dam [9,54]. The causal role of the hormone itself has been tested directly: sows given cortisol orally during gestation produced offspring with lower birth weight and an attenuated adrenocortical response to ACTH [81], which places maternal glucocorticoid exposure upstream of the offspring phenotype rather than merely alongside it. Adrenocorticotropic hormone administration to restrained pregnant sows likewise altered the HPA axis of their offspring [82], and stimulation studies in pregnant gilts have characterized cortisol dynamics in the dam and fetus separately, which is a precondition for interpreting any prenatal stress design [83]. Maternal nutrition acts on the same axis, with fish oil supplementation in late gestation and lactation affecting offspring growth, fecal microbiota structure, and post-weaning diarrhea [84], and beta-glucan supplementation improving piglet performance while maintaining sow immune status [85].

Health status is a further modifier that is easily overlooked. Serum cortisol around parturition differs between clinically healthy sows and those developing postpartum dysgalactia syndrome [86], and salivary and serum adenosine deaminase activity, reflecting the immune status, changes around farrowing in the same comparison [87]. Disease and stress are therefore not independent explanations for an elevated cortisol value, and a monitoring program that does not record health events will misattribute part of what it measures. Growth outcomes in the litter carry related information: salivary stress biomarkers were elevated in suckling Iberian piglets with lower postnatal growth independently of maternal welfare status [88].

### 1.2. Degrees of Invasiveness

Non-invasiveness is often treated as a binary property, but collection procedures used in pig research fall along a gradient, and the position of a method on that gradient determines how much of the measured signal is attributable to the sampling itself.

Three terms are used in a specific sense throughout this review and should be distinguished at the outset. Non-invasive refers to collection that requires neither restraint nor contact with the animal: urine voided onto a clean floor, feces collected after defecation, and oral fluid obtained from a chewing rope. Minimally invasive refers to collection that requires brief restraint or contact but no puncture, which covers hair clipping in every case and individual saliva sampling whenever the animal has to be held. Invasive refers to venepuncture and to procedures requiring sedation. The distinction is not merely terminological, because it determines both the additional load imposed by the measurement and the analytical unit obtained: oral fluid from a shared rope is a group-level sample and is not equivalent to saliva collected from an identified animal, although the two are frequently reported under the same heading. Hair is treated alongside the other three matrices because its time window has no alternative among them, but it is minimally invasive by the definition above, and the title of this review has been framed accordingly.

Venepuncture is invasive by any definition: the animal must be caught, restrained, and punctured. Pharmacological challenge protocols compound this, since repeated ACTH administration requires repeated handling; three-day administration in boars raised cortisol and altered immune measures [89], and stimulation in pregnant gilts has been used to characterize cortisol dynamics in dam and fetus [83]. The consequence for cortisol is not incidental but central, since restraint elicits an adrenocortical response within minutes [7,19]. Nose-snare restraint increased both serum and salivary cortisol [19]. Measurements collected during or after restraint may therefore combine the animal’s preceding state with its response to handling; less invasive collection does not make salivary cortisol insensitive to that response.

Reproductive interventions illustrate the same principle in a more pronounced form. Transvaginal ovum pick-up in sows, performed under sedation, was accompanied by measurable welfare costs and by endocrine and behavioral changes attributable to the procedure rather than to the animal’s baseline condition [71]. Where invasive sampling is unavoidable, an indwelling catheter placed well in advance remains the only way to separate the two, and this is how the classic gestational profiles were obtained [21].

Hair sampling occupies an intermediate position. Clipping a patch of neck hair causes no lasting harm and takes seconds, but the animal must still be caught and held; therefore, the procedure itself is a brief stressor. This does not contaminate the measurement in the way that restraint contaminates a plasma sample, because hair cortisol is thought to reflect a period preceding collection rather than the moment of collection [29]. The practical consequence is the reverse of the usual concern: the sampling event is not expected to affect the analyte, which is why hair is of interest as a potential indicator of longer-term exposure.

The minimally invasive category also covers procedures in which a sample is obtained during handling that would occur anyway, for instance at weighing, vaccination, or loading. Collecting at these moments reduces the additional burden to near zero, but it introduces a systematic association between sampling and an acute stressor, which must then be accounted for in the analysis rather than ignored.

Saliva obtained with a chewing rope, urine collected from a clean floor after spontaneous voiding, and feces collected after defecation involve no restraint, no puncture, and, in the case of urine and feces, no interaction with the animal at all. Rope sampling is a partial exception in that it requires the animal to engage with the device, but pigs treat the rope as enrichment; therefore, the procedure is at worst neutral and may be mildly positive [35].

Two caveats attach to the non-invasive category. First, the absence of restraint does not make the observer invisible: human presence in the pen is itself a stimulus, and the endocrine response to an unfamiliar person is well documented in pigs [90]. Protocols should therefore specify how long observers were present before collection began. Second, individual identification is harder without handling, which is why urine and feces are frequently collected at pen level rather than from identified animals; the resulting loss of individual resolution is a real analytical cost, not a technicality.

### 1.3. Scope and Aims

This review therefore treats saliva, urine, feces, and hair not as interchangeable options but as four instruments with different resolving power. Each is evaluated as a comparative monitoring tool, that is, a method whose value lies in identifying where physiological load is elevated across large numbers of animals, to be followed by more resolved measurement where a specific mechanism is at issue; the sense in which the term screening is used here is defined in Section 5.2. We examine the physiological basis of each excretory route, the analytical requirements that determine data quality and the extent to which the porcine literature meets them, the evidence base in fattening pigs and sows, the limitations that constrain interpretation, and research priorities.

## 2. Materials and Methods

The literature search was performed in Web of Science (Core Collection), Scopus, and PubMed, without a lower date restriction, that is, from database inception to July 2026 (final searches run on 24 July 2026 by L.K.; study selection by M.E.), and was supplemented by manual screening of the reference lists of all included articles and of relevant reviews. No lower date limit was applied because several of the methodological foundations of the field, including the validation of urinary and salivary cortisol in pigs and the characterization of the porcine circadian profile, date from the 1990s and remain the primary sources for statements made here [20,23,60,91].

The following search string was applied across title, abstract, and keyword fields: (“salivary cortisol” OR “urinary cortisol” OR “faecal glucocorticoid metabolite” OR “fecal cortisol metabolite” OR “hair cortisol”) AND (“pig” OR “swine” OR “sow” OR “gilt” OR “finishing pig”) AND (“stress” OR “welfare” OR “HPA axis”). It was implemented as a topic search (TS=) in Web of Science, as a TITLE-ABS-KEY search in Scopus, and with the [tiab] and [ot] field tags in PubMed. Additional searches used the terms “ACTH challenge pig”, “cortisol creatinine ratio”, “non-invasive stress biomarker swine”, and “cortisone cortisol ratio pig” to capture validation and the comparative-matrix literature.

Primary porcine biomarker studies were included if they (i) reported original cortisol or glucocorticoid metabolite data from domestic pigs; (ii) specified the biological matrix and the analytical method; and (iii) addressed methodological validation, reference concentrations, or a defined stress challenge. Primary biomarker studies were excluded if they (i) reported only plasma cortisol obtained under manual restraint without an accompanying non- or minimally invasive measure; (ii) concerned species other than the domestic pig, except where cited explicitly for cross-species comparison; or (iii) were not available in English or Hungarian. Information was extracted on study design, animal category, sample size, matrix, collection method, storage protocol, assay type and its validation status in pigs, whether normalization was applied, and the reported concentrations or response magnitudes. Reviews and studies of stress physiology, husbandry, and complementary indicators were also used to provide interpretive context. The synthesis is narrative: no review protocol was registered, and no formal risk-of-bias scoring or quantitative pooling was undertaken. The search was iterative, with terms and sources added as the review developed, and records were screened without a formal screening log. The numbers of records identified, screened, assessed, excluded, and included at each stage were therefore not recorded; they are not reported here and have not been reconstructed retrospectively. All sources used are listed in the References. Heterogeneity in matrices, assays, animal categories, and challenge designs limits direct comparisons. Assay-specific analytical and biological validation is appraised in Section 4.4, together with the design features and principal limitations of the studies supporting the main recommendations; neither appraisal replaces a uniform evidence-grading scheme.

## 3. Biological Basis and Time Windows of the Four Matrices

### 3.1. The Free Fraction as the Common Origin

Perception of a challenge activates hypothalamic corticotropin-releasing hormone (CRH) secretion, which drives pituitary ACTH release and, in turn, adrenocortical cortisol synthesis. Circulating cortisol mobilizes energy substrates, modulates immune function, and exerts negative feedback at pituitary and hypothalamic levels [4,7,8]. The magnitude and duration of the response depend not only on the stressor but on the individual: pigs differing in coping style show divergent behavioral, neuroendocrine, and immune responses to the same challenge [92], which is one reason population-level thresholds perform poorly in this species.

Cortisol circulates predominantly bound to corticosteroid-binding globulin, and only the unbound fraction is biologically active and available for diffusion into saliva, for renal filtration, and for hepatic metabolism [8,19]. This single fact organizes everything that follows: the four matrices sample the same free fraction at different points along its onward path, and therefore over different time windows. Under repeated challenge, the regulatory set-point itself shifts, with increased hypothalamic CRH and vasopressin expression accompanied by reduced glucocorticoid receptor expression [36], one mechanism by which chronic exposure alters the response to subsequent acute events.

### 3.2. Divergence of the Four Routes

Saliva receives free cortisol by passive diffusion across the acinar epithelium; therefore, salivary concentration tracks the free plasma fraction with a lag of minutes. Urine receives both free cortisol and hepatic conjugates by glomerular filtration, and these accumulate in the bladder; therefore, a spot sample integrates over the period since the previous void. Fecal glucocorticoid metabolites derive from hepatic metabolism, biliary excretion, and bacterial transformation in the gut. Fecal measurements therefore integrate excretion over gut passage and predominantly reflect metabolites rather than intact cortisol [8,23,93]. Hair is reached by a different route again. The free fraction diffuses from the capillary network supplying the follicle into the dividing cells of the hair bulb and is enclosed in the shaft as it keratinizes, with smaller contributions from sweat and sebum deposited on the emerging fiber and from local steroidogenesis within the follicle itself, which expresses a peripheral equivalent of the HPA axis [29,94]. Because incorporated cortisol is thought to be retained once keratinization is complete, hair is regarded as a potential indicator of longer-term exposure that is largely independent of the circumstances of sampling. In pigs, however, incorporation kinetics remain incompletely characterized, and a correspondence between individual segments and specific past periods has not been established [26,27,95]. The practical consequences of these differences for matrix selection are summarized in Section 5.2. The central point is that the four matrices are complementary rather than competing: fecal sampling cannot resolve immediate within-event dynamics, whereas repeated, standardized salivary sampling can contribute to studies of longer-term housing effects.

These differences have practical consequences for sampling. Saliva permits repeated measurements within a day and group-level oral-fluid collection with chewing ropes. Urine and feces can be collected after spontaneous excretion without restraining the animal, although attribution to an individual requires appropriate observation and identification. Hair is convenient to store and potentially represents a longer exposure period, but its temporal interpretation remains uncertain in pigs. The challenge-validation evidence summarized in Section 4.4 should guide matrix selection; a negative hair result alone does not establish low adrenocortical load.

**Figure 1:**
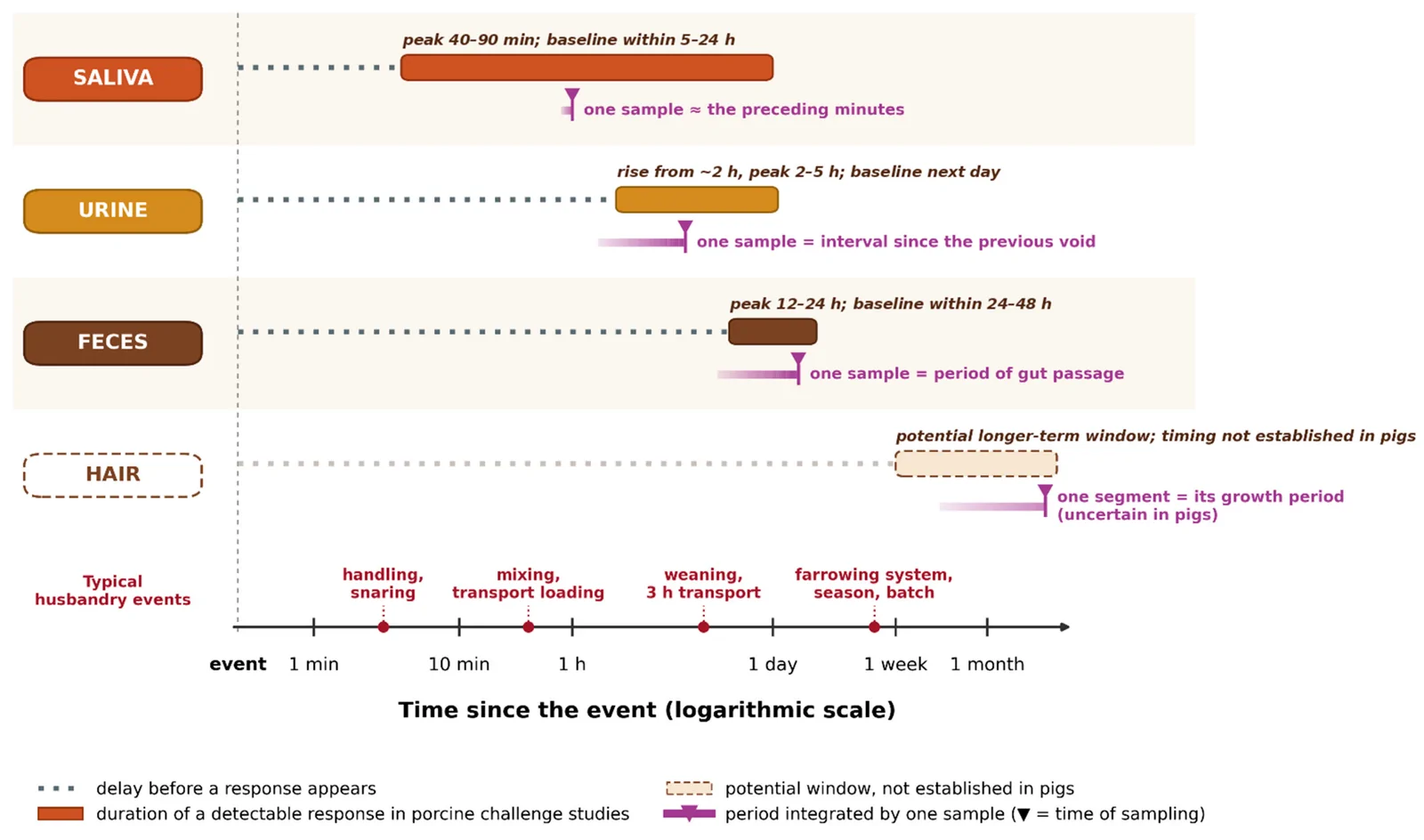
Approximate timing of the cortisol signal in the four matrices after a single event, on a logarithmic time axis. For each matrix, the dotted line marks the delay before a response appears and the colored bar the duration over which a response has remained detectable in the porcine challenge studies cited (saliva [24]; urine [21,22]; feces [24,25]). The purple marker indicates a sampling time (▼) and the period integrated by that single sample; the fading shows that this period varies in length. For hair, dashed outlines mark a potential longer-term window whose timing has not been established in pigs. Durations depend on dose, stressor, sampling, and physiology and are approximate rather than fixed properties; hair clipping is minimally invasive when restraint is required. Typical husbandry events are marked above the axis at the approximate timescale on which they act.

Because the four windows overlap only partly, a stressor that is conspicuous in one matrix may be invisible in another. Figure 1 distinguishes, for each matrix, the delay after an event before a response appears, the period integrated by a single sample, and the duration over which a response has remained detectable in porcine challenge studies, together with typical husbandry events, and can be used as a first orientation when a study is being designed.

Two qualifications attach to Figure 1. The intervals shown are central tendencies drawn from the studies cited, not fixed properties of the matrices. Urinary integration depends on the voiding interval and therefore on water intake, feeding regimen, and ambient temperature; the fecal lag depends on gut passage time, which varies with diet, age, and the stressor itself; and any hair window would depend on the growth phase of the follicle, the body site sampled, the length of the segment analyzed, and an incorporation mechanism that remains incompletely characterized [27,29,95]. The figure should therefore be read as an approximate comparison of timescales between saliva, urine, and feces, with hair shown as a potential but unestablished longer-term window, and not as a specification of the temporal resolution of any matrix.

A terminological consequence follows, and it is observed throughout the remainder of this review. What is measured in feces is not cortisol but its metabolites following hepatic and intestinal transformation, and it is referred to as fecal glucocorticoid metabolite concentration wherever it appears in the text, the tables, and the figures; fecal values are therefore not directly comparable with circulating or salivary cortisol concentrations. Salivary and hair measurements are of cortisol itself, and urine contains free cortisol together with its hepatic conjugates, for which creatinine normalization helps account for dilution. Between-study comparisons additionally require compatible assays and sampling conditions.

## 4. Analytical Requirements and the Evidence for Each Matrix

The preceding section described where cortisol goes. This one addresses how it is measured, because the analytical decision determines what a reported concentration means at least as strongly as the choice of matrix does. Two studies using the same matrix, the same species, and the same sampling protocol can differ by an order of magnitude for purely analytical reasons, and this is a major source of the between-study variability documented in Section 5.1.

### 4.1. Assay Platforms

Three families of methods are in routine use. Radioimmunoassay (RIA) is the oldest, and although it remains accurate, it has largely been abandoned because of the handling and disposal requirements of radioactive tracers. Enzyme immunoassay (EIA) and enzyme-linked immunosorbent assay (ELISA) are now the default in animal welfare research: they are inexpensive, high-throughput and require modest equipment, and validated protocols exist for porcine saliva [96]. Chemiluminescent immunoassay (CLIA) offers automation and shorter turnaround, and has been validated for porcine salivary cortisol [97], which makes large sample series feasible without the batch-to-batch variability of manual plate assays. All three are antibody-based, and therefore share one vulnerability: they report whatever binds the antibody, not necessarily the analyte of interest.

Liquid chromatography coupled to tandem mass spectrometry (LC-MS/MS) belongs to a different category. It separates compounds physically before identifying them by mass; therefore, structurally similar steroids are distinguished rather than summed. It is now regarded as the reference method for steroid quantification and permits cortisol, cortisone, and corticosterone to be measured simultaneously in a single run [98]. Its drawbacks are cost, instrument access and the analytical expertise required, which is why immunoassay remains the practical choice for most livestock studies. The appropriate role of LC-MS/MS in swine welfare research is therefore not to replace immunoassay but to calibrate it: a subset of samples analyzed by both methods establishes how far a given immunoassay departs from the true concentration in that matrix and that species.

### 4.2. Why Antibody Choice Is Not a Technical Detail

The scale of the problem is easily underestimated. Jewgenow and colleagues measured hair glucocorticoids in six mammalian species using two enzyme immunoassays that differed only in the antibody raised against the cortisol molecule, one directed against cortisol-3-CMO and the other against cortisol-21-hemisuccinate [98]. The second assay returned concentrations 2.3 to 12 times higher than the first in the same hair extracts. Chromatographic separation showed why, since up to 70% of the immunoreactivity measured by the less specific assay came from unidentified non-polar compounds that were not glucocorticoids at all. Comparison against LC-MS/MS confirmed that even the better assay overestimated the true content by up to threefold, and that in one species neither assay correlated with the reference method.

Three consequences follow for the porcine literature. First, absolute concentrations from studies using different immunoassays are not comparable, which is one reason the reference intervals discussed in Section 5.1 vary widely. The second is that an immunoassay may produce internally consistent, statistically significant results that nevertheless do not track adrenocortical activity, because the compound driving the signal is not cortisol. The third is that cross-reactivity with non-target compounds can inflate the measured immunoreactive signal and may therefore overestimate the target analyte or distort group comparisons. It does not, by itself, establish the direction or magnitude of bias in any inference about stress load, which also depends on biological variation in the cross-reacting compounds, matrix effects, and the relationship between the assay signal and HPA activity (see also Section 5.2).

### 4.3. What Validation Requires

Validation is often reported as a single sentence stating intra- and inter-assay coefficients of variation (CVs). That is precision rather than validity, and the two are independent, since a systematically wrong assay can be extremely precise. Full validation has an analytical and a biological component, and both are necessary [93,99].

Analytical validation establishes that the assay measures what it claims to in that particular matrix. It comprises cross-reactivity characterization against structurally related steroids, notably cortisone and corticosterone, parallelism testing to demonstrate that serial dilutions of the sample extract run parallel to the standard curve, spike-recovery testing, and determination of the limits of detection and quantification. Chromatographic characterization of the immunoreactive fraction, as applied by Jewgenow et al. [98], is the most stringent version of the cross-reactivity test and is the only way to discover that most of the signal is coming from elsewhere.

Biological validation establishes that the measured value responds to adrenocortical activity in the target species. The standard approach is a pharmacological challenge with ACTH, ideally combined with dexamethasone suppression to demonstrate that the signal falls as well as rises, or a naturalistic challenge of known magnitude such as transport. The porcine fecal literature reviewed in Section 4.7 is the clearest illustration of why this step cannot be skipped: an assay that had passed analytical validation in one species failed biological validation in the pig, and two decades of doubt about the matrix followed [23,24].

Table 1 sets out the minimum that a study should report for its cortisol data to be interpretable by others. The list is deliberately short, and every item is information the authors already possess at the time of analysis.

### 4.4. Validation Status of the Assays Applied in Pigs

The criteria set out in Table 1 can be applied to the porcine literature itself. Table 2 records, for the studies on which the matrix-specific conclusions of this review rest, which assay was used, whether analytical validation was reported in the porcine matrix concerned, and whether biological validation was demonstrated by pharmacological or naturalistic challenge in pigs. Analytical criteria are appraised by platform: parallelism, antibody cross-reactivity, and recovery for immunoassays, and separation selectivity, interference, calibration, extraction recovery, precision, and detection and quantification limits for chromatographic methods. Three patterns emerge. First, biological validation in pigs by pharmacological challenge is documented for saliva and, since the introduction of a group-specific assay for 5α-3β,11β-diol metabolites, for feces; for urine it rests on ACTH and CRH challenges in catheterized or group-housed sows; and for hair, repeated pharmacological stimulation has not consistently increased cortisol, although sustained housing contrasts have produced differences. Second, analytical validation is reported far less consistently than biological validation: intra- and inter-assay CVs are given by most studies, but, for immunoassays, parallelism, recovery, and cross-reactivity in the porcine matrix are reported by almost none; the studies applying the chromatographic urinary method do not restate its selectivity, calibration, extraction recovery, or detection limits, and several studies defer the question entirely to a method paper in another species or another matrix. Third, several influential studies cannot be appraised at all on this criterion because the assay is identified only by manufacturer or not at all. Taken together, these three patterns are the strongest argument for the reporting standard proposed in Table 1.

The biological validation of salivary cortisol in pigs rests on one pharmacological and one naturalistic protocol. In the pharmacological case, four gilts received an ACTH analog at approximately 10 µg/kg intramuscularly and two received saline; salivary concentrations rose by a median of 1100% above baseline in the treated animals, peaked 40 to 90 min after injection, and returned to baseline within 5 to 24 h [24]. The saline controls nonetheless rose by a median of 400%, which is a reminder that handling alone moves this matrix and that a sham-treated group is not optional in such a design. The response is reported descriptively, without inferential statistics. In the naturalistic case, four hours of social isolation applied to growing pigs at 12 and at 20 weeks of age produced a salivary response whose magnitude depended on the time of day at which the isolation began (*p* < 0.05), in the study that also characterized the porcine circadian profile [3].

For urine, the evidence is the most complete of the four, because it includes both suppression and stimulation. In ten pregnant sows carrying jugular and indwelling bladder catheters, dexamethasone at 20 µg/kg intravenously reduced nocturnal urinary cortisol from 13.0 to 2.6 pg per µg creatinine (*p* < 0.0001), and ovine CRH at 1 µg/kg intravenously raised plasma cortisol tenfold within 30 min and urinary cortisol from 1.7 to 30.0 pg per µg creatinine, with the urinary peak 2 to 3 h after injection [21]. A depot preparation of ACTH given intramuscularly to ten pregnant sows, against ten saline controls, raised urinary cortisol from about 2 h after injection, with a peak between 2 and 5 h and a return to baseline the following day [22]. The earliest of these studies validated the matrix differently again, by contrasting breeds of known differing adrenocortical activity rather than by challenge: Meishan sows excreted almost five times as much cortisol per unit creatinine as Large White sows (*p* < 0.0001), a difference already established in plasma [20]. Together, these define both the direction of the urinary response and its lag, which is the information a sampling protocol requires.

The fecal record is the one in which validation status changed the conclusion, as Section 4.7 sets out. The 11-oxoaetiocholanolone assay that performs well in ponies detected an increase in only three of six pigs after 0.75 mg of ACTH intravenously, and showed no suppression after 5 mg of dexamethasone [23]. The group-specific assay for 5α-3β,11β-diol metabolites, applied to the same species, gave a median increase of 180% after challenge with a peak at 24 h [24], and detected the response of 36 fattening pigs to a three-hour road journey, with a treatment by time interaction of F(3,107) = 2.71, *p* = 0.049, a peak 12 to 24 h after the end of the journey, and a return to baseline within 24 to 48 h [25].

Hair is the matrix for which controlled validation has been attempted and has not succeeded. Fourteen intramuscular injections of an ACTH depot preparation, given over four weeks to 38 gilts, produced no increase in native hair (*p* = 0.10), in regrown hair, or in hair segments, and in the segments the untreated controls were in fact higher (*p* < 0.05); cattle treated identically in the same experiment responded in all three hair types (*p* < 0.001) [26]. A positive porcine result comes from a sustained husbandry contrast rather than a pharmacological one: 21 days of crowding with repeated mixing raised hair cortisol from 75.6 to 87.3 pg/mg in 24 artificially reared piglets (*p* = 0.005), while salivary cortisol sampled from the same animals did not differ (*p* = 0.447) [27]. Heat stress produced the same asymmetry in another form, hair cortisone and the cortisone-to-cortisol ratio rising in uncooled rooms while hair cortisol itself did not change [30]. These contrasting results do not establish a predictable temporal response for porcine hair cortisol. They support cautious interpretation alongside the broader uncertainty identified in a systematic review across vertebrate species [95].

Table 3 complements this assay-level appraisal with the design features of the same studies, namely control groups, experimental units, repeated measurements, and confounding, which determine how far their results can support the recommendations made here.

### 4.5. Saliva

Salivary cortisol is the free, unbound fraction, which gives the matrix an interpretive advantage the other three lack: the measured analyte is the biologically active species rather than a surrogate for it. In pigs it represents 8.6–13.3% of the serum concentration [19]. The proportion depended on concentration: pooled across ACTH and nose-snare challenges, it was 13.3% in pre-stimulation and 8.6% in maximal samples, and after ACTH the salivary increment (164%) was smaller than the serum increment (448%), whereas after nose-snaring it was larger (236% against 124%) [19]. Because diffusion is passive and largely flow-independent, the concentration is not diluted by increased secretion in the way that urinary concentration is diluted by water intake. The response magnitude is correspondingly large. In female pigs subjected to ACTH challenge, salivary glucocorticoid concentrations rose by approximately 1100% [24]—an order of magnitude greater than the fecal response measured in the same animals. This supports its use for acute-response monitoring, but response amplitude in one experiment does not establish superior diagnostic sensitivity across matrices.

Saliva is obtained by allowing animals to chew an absorbent device, most often a cotton rope suspended in the pen, and this apparently trivial step carries consequences. Collection strategy and animal age both influence measured analyte concentrations [102], and devices differ in recovery; therefore, the protocol must be specified and held constant. The sampling method itself has been compared systematically for porcine saliva, with consequences for both the concentrations obtained and their usefulness in welfare assessment [103]. The rationale for the matrix and the analytical requirements it imposes have been set out in detail for pigs [17]. Immunoassays developed for human saliva have been validated for porcine saliva [96], and a full profile of salivary analytes across a growing-finishing cycle is available as a reference framework [104]. Rope sampling has the further advantage that pigs treat the rope as enrichment; therefore, collection is close to stress-free and can be applied at group level [35].

The dominant constraint is timing. Salivary cortisol in growing pigs follows a pronounced circadian rhythm modulated by age, sex, weight, temperature, and behavior [3,105], and the amplitude is sufficient that an unrecorded shift of one to two hours in collection time can generate differences comparable to those attributed to treatment. The rhythm was described in young pigs three decades ago and has been confirmed repeatedly since [91]. Studies that do not report clock time cannot be evaluated on this point. On the analytical side, several immunoassay platforms have been validated for porcine salivary cortisol; among them, an automated chemiluminescent system makes high-throughput measurement feasible without the batch effects of manual plate assays [97].

Saliva has the broadest evidence base of the four. Psychosocial challenge alters salivary biomarkers of stress and immunity [106]; handling conditions at slaughter are discriminated by salivary analytes [70]; and endocrine responses to a range of common stressors have been compared across indicators in gilts [90]. Of particular relevance here, salivary cortisol, cortisone, and 11β-hydroxysteroid dehydrogenase type II (11β-HSD2) activity have been characterized across pregnancy and lactation in sows [107]. A complementary sialochemistry study followed a wider panel of saliva analytes through pregnancy, farrowing, and lactation in the same species [108], and salivary biomarkers have been shown to track litter-level outcomes, being elevated in lactating piglets with lower postnatal growth independently of the welfare status of the dam [88].

A recurring finding is that the cortisone-to-cortisol ratio may outperform cortisol alone under sustained load, because 11β-HSD2 activity increases during chronic challenge [30,70,107]. This is a promising refinement rather than an established endpoint. Salivary cortisol has also proved sensitive enough for herd-level comparison of husbandry systems, discriminating gestation feeding systems across commercial herds [49] and detecting transport-induced change in parallel with heart rate variability [74]. The limitations of interpreting these responses without complementary markers and behavioral observations are considered in Section 5.3.

### 4.6. Urine

Urine contains free cortisol and, predominantly, glucuronide and sulfate conjugates. The conjugated forms are more stable than free cortisol under practical storage conditions, which is an underappreciated advantage of the matrix [20]. Validation in pigs dates to Hay and Mormède, who showed that urinary cortisol excretion separates breeds of known differing adrenocortical activity, Meishan sows excreting almost five times as much cortisol per unit creatinine as Large White sows [20]; the approach was subsequently extended to pregnant sows under dexamethasone and CRH challenge [21] and to a housing comparison under ACTH challenge [22] and to a comparison of malignant hyperthermia genotypes in barrows housed in metabolic cages [109].

Because cortisol accumulates in the bladder, a spot sample yields an integrated rather than instantaneous value. The integration window is not fixed, however: it is the interval since the previous void, which is neither controlled nor usually known.

Spontaneous voiding collected from a clean floor surface is fully non-invasive and permits high sample numbers. Collection during morning penning increases yield but introduces a systematic association with the circadian peak. Either way, the collection window must be fixed and reported. Where the design permits, sampling before the morning feed is preferable, since feeding alters HPA activity and feed anticipation in restricted-fed animals is itself a documented stressor [11].

Samples should be centrifuged to remove particulates and stored at 4 °C for short periods or −20 °C for longer intervals; repeated freeze–thaw cycles reduce recovery and must be avoided.

Concurrent creatinine measurement corrects for urine dilution and provides a stable reference, since creatinine production is proportional to muscle mass [20]. In catheterized sows in which urine was collected continuously, creatinine-corrected concentrations tracked the directly measured excretion rate closely, with correlations of 0.90 for cortisol and 0.92 for cortisone, and concentrations in night urine predicted the 24 h excretion rate with correlations between 0.65 and 0.93 [21]. The validated porcine method is solid-phase extraction followed by high-performance liquid chromatography (HPLC) with ultraviolet detection [101], and the studies on which this section rests all use it [20,21,22]. Expressing results as a cortisol-to-creatinine ratio is standard in the literature. Uncorrected concentrations in ng/mL are therefore not directly comparable with ratio-based studies, and the omission is substantive rather than cosmetic. Feeding management is a further determinant that is easily overlooked in finishing pigs, where feed texture, meal frequency, and pre-slaughter fasting all affect urinary cortisol alongside carcass and meat quality [110].

A kinetic consideration constrains sampling design: following depot ACTH administration, urinary cortisol increased from approximately 2 h after injection and peaked between 2 and 5 h (mean 3 h 40 min), returning to basal values 9–24 h after injection; no circadian variation was detected on the preceding day [22]. These intervals describe time after administration, not a directly measured lag behind plasma cortisol. Protocols intended to capture a specific challenge must account for both response kinetics and the voiding interval.

### 4.7. Feces

**Figure 2:**
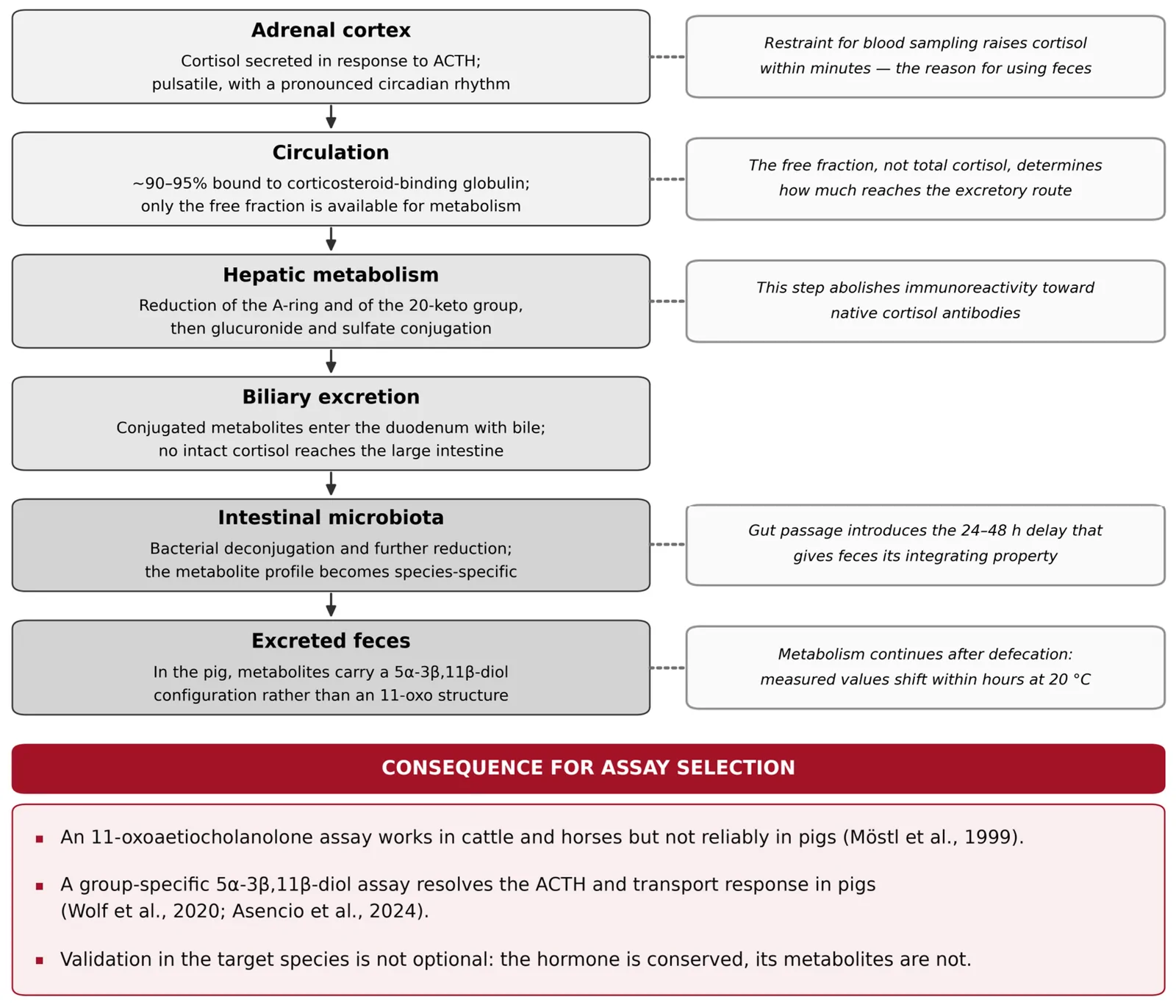
Successive transformation of cortisol on the biliary–intestinal route, and why an assay validated in one species need not transfer to another. Compiled from Möstl et al. [23], Palme et al. [111], Wolf et al. [24], and Asencio et al. [25]. ACTH, adrenocorticotropic hormone.

The fecal route is the most transformed of the four, and this is simultaneously its principal strength and its principal pitfall. Cortisol is metabolized in the liver by reduction in the A-ring and of the 20-keto group, then conjugated and excreted in bile. In the intestine, bacterial enzymes deconjugate and further reduce these compounds. What is eventually excreted is therefore not cortisol but a species-specific family of metabolites, and the immunoassay must be chosen to recognize that family (Figure 2).

The evidence for this is direct. Following infusion of 14C-labeled cortisol into ponies and pigs, HPLC combined with three enzyme immunoassays showed that only an 11-oxoaetiocholanolone assay co-eluted with radioactivity, and biological validation gave discordant results between species: in horses, ACTH produced a rise exceeding 200% above baseline and dexamethasone produced a fall, whereas in pigs an increase was detected in only three of the six animals tested after ACTH, and dexamethasone produced no decrease at all [23]. The mechanistic explanation the authors offer is that only about 7% of infused cortisol is excreted in the feces of pigs, against 41% in ponies and 28% in sheep, such that the porcine fecal signal is both smaller and more variable in its lag [23]. The small size of that validation group is itself relevant, since a response present in half of six animals is compatible both with a genuinely weak effect and with an assay that fails to recognize the relevant metabolites. For two decades this discordance was read as evidence that fecal glucocorticoid measurement was simply unreliable in pigs.

The reinterpretation came with assay selection rather than with improved sampling. A group-specific enzyme immunoassay measuring metabolites with a 5α-3β,11β-diol configuration resolved the porcine response cleanly, showing a 180% increase after ACTH administration and a 70% increase after transport [24]. The same assay subsequently detected the response of fattening pigs to a three-hour road journey and the accelerated recovery produced by dexamethasone administration [25]. These results indicate that assay selection was an important contributor to the earlier inconsistency. They do not show that it was the sole cause, because the evidence comes from studies with different designs, animals, and challenges rather than from a fully validated comparison under common conditions; the low fecal excretion of cortisol in pigs, the variable delay, the small early sample, and individual variability may also have contributed [23,24,25].

This episode has a general lesson that extends well beyond pigs: physiological validation in the target species is not an optional refinement but a precondition for interpretation, because the hormone is conserved while its excreted metabolites are not [93,112].

Collection after defecation requires no restraint or cooperation from the animal, and it is less affected by circadian and episodic fluctuation than saliva or urine [8,113]. Samples can be collected from the floor and repeated over time without disturbing the animals. One qualification must be stated explicitly. Growing and finishing pigs are group-housed; therefore, a sample taken from the pen floor cannot generally be assigned to a known individual unless defecation is observed and the animal identified. Under group housing, the realistic unit of analysis is therefore the pen rather than the pig, and fecal sampling addresses the group-level question of how a batch responds to a housing or management factor. Individual-level fecal profiling remains restricted to gestating sows in stalls, to experimental settings, or to protocols in which defecation is observed directly and the animal identified at that moment.

The counterweight is post-defecation instability. Bacterial metabolism continues after excretion, and measured metabolite concentrations in porcine feces increase significantly after 24 h at room temperature [23]. Reported stability windows differ between studies: using the 5α-3β,11β-diol assay, changes remained within approximately 12% over about two days under natural conditions [24]. The discrepancy is most plausibly explained by assay specificity, since different metabolite classes degrade at different rates. In practice, this means that collection must be prompt, the interval between defecation and freezing must be recorded, and thawing conditions must be standardized [23].

Comparative work relating plasma, saliva, urinary, and fecal glucocorticoid metabolite measurements in pigs provides an additional basis for evaluating temporal differences between matrices [100].

The smaller fecal response than the salivary response in the paired ACTH experiment [24] should not be interpreted as diagnostic inferiority. Temporal integration, sampling intervals, and assay performance must be considered before comparing response magnitudes.

### 4.8. Hair

Cortisol is incorporated into the hair shaft during growth, principally by diffusion from blood into the follicle; therefore, hair is considered a potential indicator of exposure over the preceding weeks to months [29]. It offers the longest potential window of the four matrices, but in pigs the correspondence between a given segment and a specific past period has not been established (Section 4.4). Reported concentrations in pigs are low in absolute terms, on the order of a few picograms per milligram; therefore, analytical sensitivity is a practical constraint [31]. Its potential suitability as a stress indicator in this species was investigated in a dedicated methodological study [114].

A cortisone fraction is measurable alongside cortisol, and the ratio between the two has proved more informative than cortisol alone under thermal load: growing pigs reared at high ambient temperature showed changes in the ratio that cortisol by itself did not resolve [30]. The same pattern appears in saliva [70,107], which suggests that 11β-HSD2 activity, rather than glucocorticoid output alone, carries part of the chronic-stress signal.

Hair is clipped from a standardized site, most often the neck, and the animal must be restrained briefly (Section 1.2). Two preanalytical steps then dominate the result. The first is washing: external contamination with feces, urine, or dust can add glucocorticoid to the sample surface, and the wash protocol determines how much of it is removed. Pollock and colleagues showed experimentally that both contamination level and wash procedure alter measured cortisol and dehydroepiandrosterone concentrations in swine hair, which means that protocols differing in this step are not directly comparable [28]. The second is segmentation: because different segments grew at different times, the length of hair analyzed defines the exposure window, and unsegmented whole-length samples average across periods that may differ substantially. The remedy is to segment the hair by defined length before extraction, to record both the segment length and the interval since the previous clipping, and, where the question concerns a specific period, to shave the site in advance and analyze only the regrown material, such that the growth window is set by the investigator rather than inferred afterward.

Growth rate itself is variable. Season, body site, sex, and breeding system all affect hair cortisol in pigs; in mixed organic herds these factors accounted for a substantial share of the observed variation [33]. The determinants have been examined systematically in pigs and cattle together, and coat color, body region, and sampling interval each contribute measurable variation [94,115]. Sunlight exposure is a further confounder in outdoor systems.

The pharmacological and housing studies summarized in Section 4.4 yielded contrasting hair cortisol responses [26,27]. A systematic review across vertebrate species, which included no porcine studies, also questioned a simple interpretation of hair glucocorticoids as a retrospective index of long-term stress [95]. Neither this cross-species synthesis nor the porcine challenge studies establish a universally applicable exposure window for pigs.

Field studies give a similarly qualified picture. Sows housed in farrowing crates had numerically higher hair cortisol than sows in loose housing, but the difference was not significant, and the authors noted that coexisting dominant stressors such as farrowing itself and the presence of suckling piglets may mask a housing effect [31]. Across twenty commercial farrow-to-finish farms, hair cortisol in finishing pigs varied more between individuals within a batch than between farms, which sets a practical floor on how few animals can be sampled if a batch is to be characterized at all [32].

Against this, one intervention study did produce a clear result: shortening the period for which sows were restrained during lactation improved production and lowered hair cortisol in both the sows and their piglets [116]. This finding supports further investigation of sustained husbandry effects but does not resolve the inconsistent challenge-validation results. Reproductive cycling provides a further test of that window: hair cortisol followed across two consecutive reproductive cycles in sows varied with cycle stage rather than remaining constant [117], and hair cortisol during gestation has been examined alongside the blood cortisol profile at parturition in the same animals [118]. At the end of the production cycle, hair cortisol sampled at slaughter has been evaluated together with haptoglobin and C-reactive protein as a retrospective indicator of lifetime welfare status [119]. The practical position is therefore that hair should be treated as an exploratory rather than an established indicator in pigs. It is the only matrix that may reach back over weeks, which makes it of potential interest for questions about housing system, season, or batch; but the assay has not been validated against controlled challenge in this species with the consistency achieved for saliva and feces, and negative findings cannot currently be interpreted as evidence of low stress.

## 5. Cross-Matrix Comparison and Decision Framework

### 5.1. Concentrations Reported in Pigs

Reported baselines vary widely, and much of that variation is methodological rather than biological. Uncorrected urinary cortisol concentrations vary considerably among pigs. In German Landrace barrows housed in metabolic cages, concentrations in 12-h urine collections ranged from 0.6 to 94.3 ng/mL, with most measurements below 20 ng/mL [109]. Variation of this magnitude means that single uncorrected values cannot distinguish unstressed from stressed animals. For serum, the interval most often invoked is 27.5–31.8 ng/mL [34]. This figure is quoted from a textbook appendix of laboratory values and was not derived as a reference interval in the clinical-pathology sense: the reference population, the preanalytical conditions, and the assay are not specified, and no partition by age, parity, or production stage is available. It is used here as a benchmark that the literature has adopted rather than as a validated reference interval, which is consistent with the observation that most values from commercial herds fall outside it [35]. The same caution applies to the urinary figures quoted above and to the values collected in Table 4. Social challenge produces the largest documented effects, with newly mixed sows showing several-fold elevations relative to stable groups [10,41]; weaning elicits a three- to fourfold rise within 24 to 48 h [12]; and feed deprivation produces a graded response [11].

The relative-change column in Table 4 describes the direction and approximate size of each response within its source study only. It is not a basis for comparing responses between studies or matrices, either quantitatively or by order of magnitude. The studies differ in antibody, platform, units, normalization, sampling time, animal category, and stressor, and, as Section 4.2 sets out, assays differing only in the antibody employed have returned concentrations differing several-fold from identical extracts. The column therefore supports statements of the form “this stressor raised this matrix” and not statements of the form “this matrix is more sensitive than that one”.

### 5.2. Choosing a Matrix for a Given Question

The preceding sections support a practical decision framework, which should be read as a guideline derived from the evidence reviewed above rather than as a set of rules. If the question concerns an identifiable event, such as mixing, a handling procedure, transport loading, or castration, saliva is well suited, because repeated collection can capture rapid endocrine changes and its response to pharmacological and acute challenge has been demonstrated [3,24]. If the question concerns cumulative load over hours to days, such as a nutritional intervention or a production stage, urine is well suited, given its demonstrated responses to suppression and stimulation [21,22], provided creatinine correction is applied and collection time is standardized. If the question concerns chronic load in animals that must not be disturbed, feces is well suited, given its demonstrated response to pharmacological and transport challenge with a matched assay [24,25], provided the assay matches the porcine metabolite profile and samples are frozen promptly. If the question concerns exposure over weeks, for instance a farrowing system, a season, or a batch, hair is the only matrix that may reach that far back, but its response to controlled challenge in pigs has not been consistently demonstrated; therefore, the recommendation is correspondingly provisional and negative results should not be read as evidence of low load. Table 5 sets out the specifications that follow, including the degree of invasiveness defined in Section 1.2. Because no study has compared the four matrices directly under a common challenge in pigs, this framework does not rank the matrices; it matches them to temporal suitability and demonstrated applications, and it rests on cross-study inference.

What none of the four can do is distinguish a welfare-relevant elevation from a physiologically appropriate one. Gestation, lactation, and growth all alter the set-point of the HPA axis independently of any welfare insult; therefore, an elevation measured during a metabolically demanding production stage cannot be read as evidence of compromised welfare without an independent indication of what produced it. This is a limitation of scope rather than of execution, and it defines the questions these methods should be asked. The finding that oral fluid cortisol did not correlate with structured welfare scores across six commercial farms [35] should be read in the same light.

These matrices can support first-stage comparative monitoring rather than diagnosis. Group differences may identify where further assessment is warranted; behavioral observations, production records, clinical examination, and targeted sampling can then help investigate their causes. The term comparative screening is used in this restricted sense throughout this review. The evidence reviewed does not establish generalizable sensitivity, specificity, or decision thresholds for a defined welfare condition across the four matrices. Measurement repeatability and diagnostic validity are distinct and require context-specific evaluation. Category-specific reference intervals would support interpretation but would not, by themselves, validate a welfare diagnostic test.

### 5.3. What Cortisol Cannot Do, and What Other Markers Add

This review has treated cortisol as the analyte of interest throughout. That focus is defensible, since cortisol remains the most widely measured stress indicator in pigs, but it leaves an obvious question unanswered: what does cortisol not tell us, and what would have to be measured alongside it?

The central conceptual limitation is that glucocorticoid secretion is not specific to negative experience. Ralph and Tilbrook, reviewing the use of glucocorticoids in welfare assessment, emphasized that cortisol has many normal, non-stress-related functions and that its synthesis increases in response to pleasure, excitement and arousal as well as to fear, anxiety and pain; increased glucocorticoid output can accompany positive welfare states, and a stress response can improve rather than reduce fitness [120]. A rise in cortisol therefore indicates that the animal is aroused, and not necessarily that it is suffering.

This distinction is particularly relevant during lactation: the accompanying endocrine elevation may reflect normal metabolic demands [121,122,123] and does not, by itself, establish compromised welfare. Interpretation requires information on the animal’s physiological stage, behavior, and clinical condition.

If cortisol cannot distinguish the valence of arousal, it also cannot distinguish between stressors. Ott and colleagues applied several different challenges to pigs and measured cortisol, haptoglobin, and chromogranin A in saliva; the three analytes responded differently depending on which challenge was applied; therefore, no single marker ranked the stressors by severity [124]. In a lipopolysaccharide challenge model, salivary adenosine deaminase, haptoglobin and cortisol followed distinct time courses [125], which means that the interval between challenge and sampling determines which marker appears informative.

A panel therefore does more than add precision, because it adds dimensions. Cortisol supplies the magnitude and timing of HPA activation; acute phase proteins such as haptoglobin add an inflammatory and infectious dimension that distinguishes disease from husbandry stress, which matters given that serum cortisol around parturition differs between healthy sows and those developing postpartum dysgalactia syndrome [86,87]; chromogranin A reflects sympatho-adrenomedullary rather than adrenocortical activation; and the salivary cortisone-to-cortisol ratio, discussed in Section 4.5 and Section 4.8, reflects 11-beta-hydroxysteroid dehydrogenase type II activity and appears to carry additional information under chronic and thermal load [30,70,107].

Even a well-chosen panel remains a panel of arousal markers. Moscovice and colleagues exposed pigs to social challenges and found that salivary cortisol varied with the challenge whereas salivary oxytocin did not, and concluded that the endocrine measures available do not yet allow emotional valence to be inferred [126]. Behavioral and production measures are not optional supplements to endocrine monitoring; they are the part of the assessment that carries the information cortisol structurally cannot.

The missing information is behavioral. Direct observation remains the reference approach, and the structured welfare protocols used on commercial farms rest on it. That the two sources are not interchangeable is shown by the finding that oral fluid cortisol did not correlate with such protocol scores across six commercial herds [35]: the protocol scored what the animals were doing and how they were kept, while the saliva measured adrenocortical output, and the two need not move together. Behavioral assessment is nevertheless labor-intensive, requires trained observers, and captures a short window on a small number of animals, which is precisely the constraint that group-level endocrine sampling was intended to relieve.

Precision livestock farming (PLF) technologies offer a route out of that trade-off, because they observe behavior continuously and automatically. In the PLF concept, the animal itself functions as the sensor, and algorithms translate its measured responses into indicators of performance and welfare [127]. A systematic review of commercially available systems for pigs mapped which welfare indicators each technology can address, including activity, feeding, drinking, vocalization, cough, and lameness [128]. Two families are directly relevant here. Acoustic monitoring analyses vocalization and cough sounds; cough sound analysis distinguishes respiratory infection from other sounds in pig houses [129], and real-time classification of sick cough has been achieved under farm conditions [130]. Video image analysis extracts posture, locomotion and interaction from camera streams; image features have been used to classify aggressive interactions [131], and automated detection of behavioral change has been proposed for early identification of health and welfare compromise [132].

The complementarity with cortisol is structural rather than incidental, and it can be stated in the terms used throughout this review. Every matrix considered in Section 3 and Section 4 was characterized by its time window, and the recurring difficulty was that a concentration cannot be interpreted without knowing what happened inside that window. A continuous behavioral record supplies exactly that. For saliva, whose window is minutes, the PLF record identifies what the animal was doing in the minutes before the swab. For feces, whose window spans gut passage, it identifies which events fell inside the integration period and which did not. For hair, whose potential window is longer, it provides the husbandry history over which the signal was accumulated. In each case the behavioral record converts an uninterpretable number into an interpretable one.

The exchange runs in both directions, and this is the point most easily overlooked. PLF systems detect that behavior has changed, but they do not establish that the change carries a physiological cost; an algorithm that flags reduced activity cannot distinguish an animal that is resting from one that is withdrawing. Cortisol supplies that missing dimension, since it quantifies the adrenocortical load associated with the detected change. A validated behavioral alert accompanied by an elevated fecal glucocorticoid concentration is evidence of a different kind from either measure alone.

Two practical consequences follow for study design. The sampling protocol can become event-driven rather than fixed, since a continuous behavioral record can indicate when a sample is informative, which raises the proportion of samples that carry signal; and the matrix can be chosen to match the timescale of the detected behavioral change rather than by convention, following the framework in Section 5.2.

For the assessment of chronic stress in particular, this pairing is the most promising avenue currently available. Chronic load is defined by its persistence, and persistence is what continuous monitoring measures best; the endocrine matrices with long integration windows, feces and hair, operate on the same timescale. Their combination is therefore not merely additive but well matched, and we regard it as the principal direction in which the practical use of non-invasive cortisol measurement in pigs is likely to develop.

## 6. Limitations, Reporting Standards, and Research Priorities

The constraints below are not of equal weight. Two warrant particular attention: uncertain assay validity in pigs and the absence of category-specific reference intervals. The former limits confidence in the measured signal; the latter constrains threshold-based interpretation without invalidating appropriately controlled comparisons. The remainder are serious but manageable, in that they bias a result whose direction can usually be anticipated and whose magnitude can be reduced by design. We therefore treat them in that order.

### 6.1. Constraints on Interpretation

Assay–species mismatch. The fecal literature indicates that an assay validated in one species may perform poorly in another, and that assay selection was an important contributor to the inconsistent porcine results, alongside excretion kinetics, animal variability, and study design [23,24,25]. Studies that do not report the assay used and its validation status in pigs are difficult to interpret.

Dilution and normalization. Without creatinine measurement, urinary concentration conflates hormone output with water balance. Since water intake varies with ambient temperature, feed composition, and lactational stage, this is not random error but bias correlated with the variables under study.

Circadian confounding. The diurnal amplitude in pigs is large enough that a shift of one to two hours in sampling time can generate differences comparable to those attributed to treatment [3,105]. This affects saliva most and feces least.

Age, parity, and genotype. Cortisol declines modestly with age in pigs [3,39], HPA reactivity is heritable [40], and both effects are superimposed on any treatment comparison. Age-unadjusted comparisons across categories are difficult to interpret.

Absence of reference intervals. No validated species-specific reference intervals exist for any of the four matrices in pigs stratified by age, parity, and production stage. The one serum interval in common use [34] excludes most commercial values when applied in the field [35], which points as much to the inadequacy of the interval as to the welfare status of the herds.

Confounding of stage with time. In longitudinal designs, physiological stage advances together with calendar time; therefore, seasonal and management-related trends are structurally confounded with the biological variable of interest. Any comparison between production stages sampled sequentially within a single herd carries this confound, and it can be broken only by staggering entry into the study or by including a contemporaneous comparison group at each stage.

Individual variability. CVs near 50% within a single physiological stage mean that group-level comparisons have limited power unless sample sizes are large or individual baselines are established. Coping style is one documented source of this heterogeneity [92].

Preanalytical processing. Hair is the clearest case, where wash protocol and contamination level demonstrably alter the measured concentration [28], but the principle is general: the interval to freezing for feces, the collection device for saliva, and the freeze–thaw history for urine each introduce variation that is invisible in the final number unless it is reported.

Health status as a confounder. Cortisol responds to disease as well as to husbandry. Serum concentrations around parturition differ between clinically healthy sows and those developing postpartum dysgalactia syndrome [86,87]; therefore, a monitoring program that does not record health events will attribute part of what it measures to the wrong cause.

### 6.2. Research Priorities

The priorities below follow directly from the constraints just described. Paired-matrix validation and category-specific reference intervals are complementary priorities; the remaining proposals address measurement consistency and interpretation.

Paired-matrix validation. The most immediate need is for studies measuring salivary, urinary, and fecal glucocorticoids in the same animals across a defined challenge, with creatinine normalization applied to urine and a validated porcine assay applied to feces. Wolf et al. [24] provide the template for two matrices; extending it to three, with concurrent plasma sampling in a subset, would characterize relationships between matrices and quantify the lag structure empirically.

Reference intervals stratified by category. Establishing intervals for weaners, growers, finishers, gilts, gestating sows by trimester, and lactating sows by week would improve interpretation of concentrations; diagnostic use would additionally require validation against a defined target condition. The repeated finding that most commercial values fall outside the commonly cited serum benchmark [34,35] makes this the field’s most conspicuous gap. It requires multi-farm sampling with standardized protocols rather than larger single-herd studies.

Standardized sampling windows. A consensus protocol specifying collection time relative to feeding and to the circadian peak, minimum duplicate structure, mandatory reporting of clock time, and, for feces, the interval between defecation and freezing would substantially improve comparability. Variability presently attributed to biology is in part attributable to the absence of such a standard.

Individual baseline approaches. Given CVs near 50%, analyses referenced to each animal’s own baseline are likely to be considerably more sensitive than population-threshold comparisons.

Validation of hair in pigs. Hair is the only matrix that may reach back over weeks, and it is also the only one whose response to controlled challenge has not been consistently demonstrated in this species: a sustained housing challenge raised it [27], whereas repeated ACTH administration did not [26]. A dedicated validation combining repeated ACTH administration with segmented analysis, a standardized wash protocol [28], and concurrent measurement in a second matrix would establish whether the negative findings reflect the marker or the design. Until this is available, hair should be reported alongside a validated matrix rather than alone.

Dispersion as an endpoint. The substantial individual variability discussed above warrants examination of response distributions, although a high value alone does not establish impaired welfare. An intervention that narrowed the distribution of responses without shifting its center would be invisible to a mean-based analysis. Dispersion and upper-percentile measures therefore deserve to be reported alongside means as a matter of routine.

The cortisone-to-cortisol ratio. Evidence that this ratio outperforms cortisol alone under chronic load is accumulating in saliva and hair [30,70,107], but it has not been examined in urine or feces in pigs. Given that 11β-HSD2 activity is itself modulated by chronic challenge, this is a tractable and potentially valuable extension.

Nutritional modulation with adequate duration. Evidence that omega-3 and microalgal supplements modulate HPA activity in pigs remains limited [133,134,135], and the latency data on which mechanistic explanations rest derive largely from human and laboratory-animal work [136,137,138]. Dose–duration designs within a single production batch, with arms at 0, 30, 45, and 60 days, would establish whether a duration effect exists in this species and what minimum exposure it requires.

## 7. Conclusions

Saliva, urine, feces, and hair each occupy a defensible position in the assessment of stress in intensively housed pigs, and the choice between them should follow from the time window a question requires rather than from convenience alone. Repeated salivary sampling can characterize rapid responses to discrete events, and it can be collected at the group level with chewing ropes, although a rope sample is a group-level and not an individual measurement. Urine integrates secretion over the interval since the previous void and suits cumulative effects of nutrition and housing. Feces reflects excretion integrated over gut passage, after a delay that depends on gut transit, and requires no disturbance, provided the assay recognizes the porcine metabolite profile. Hair is a potential indicator of longer-term exposure, but its temporal correspondence in pigs has not been established; it is minimally rather than fully non-invasive, and its evidence base in pigs remains the weakest of the four. Because no direct comparison of the four under a common challenge has been published in pigs, these positions rest on cross-study inference rather than on head-to-head evidence.

The fecal literature carries a transferable methodological lesson. A response that appeared unreliable for two decades was detected once a group-specific assay for 5α-3β,11β-diol metabolites was applied, which identifies assay selection as an important contributor; differences in excretion kinetics, animal variability, and study design between the studies concerned remain plausible additional explanations. Physiological validation in the target species is a precondition for interpretation, not a refinement of it.

Standardized collection times, creatinine normalization for urine, species-appropriate assays, and controlled preanalytical handling would improve comparability. Important evidence gaps remain in category-specific reference intervals, hair incorporation kinetics, and direct comparisons under common challenges. Recommendations on relative matrix performance should therefore remain provisional.

These methods are most useful as components of comparative monitoring, with matrix choice matched to the expected response window. Determining whether an observed difference reflects compromised welfare requires complementary behavioral, clinical, and production measures. Cortisol measurements should guide such assessment rather than replace it.

## Figures and Tables

**Table 1:** Minimum information a study should report for cortisol measurements in pigs to be interpretable and comparable across laboratories.

Item	What Should Be Stated	Why It Matters
Matrix and fraction	Saliva, urine, feces or hair; free cortisol, conjugates or metabolites	Determines the time window and the analyte
Assay platform and supplier	RIA, EIA/ELISA, CLIA or LC-MS/MS; antibody and kit identity	Absolute values are not comparable across platforms or antibodies
Validation status in pigs	Analytical (cross-reactivity, parallelism, recovery, LOD/LOQ) and biological (ACTH challenge or equivalent)	An assay validated in another species may not transfer
Intra- and inter-assay CV	Percentages, with the number of replicates	Precision, distinct from validity
Clock time of sampling	Actual time of day, and time relative to feeding	Circadian amplitude can exceed the treatment effect
Sampling device or procedure	Rope type and chewing duration; void collection method; hair site and segment length	Device and site materially alter the measured value
Preanalytical handling	Interval to freezing, storage temperature, freeze–thaw cycles, wash protocol for hair	Post-collection change is invisible in the final number
Normalization applied	Creatinine for urine; per g feces; per mg hair; none	Uncorrected and normalized values are different quantities
Exclusions	Number and reason for samples excluded	Selective exclusion biases group comparisons
Concurrent covariates	Age, parity, production stage, health events, ambient temperature	All are documented determinants of cortisol

ACTH, adrenocorticotropic hormone; CLIA, chemiluminescent immunoassay; CV, coefficient of variation; EIA, enzyme immunoassay; ELISA, enzyme-linked immunosorbent assay; LC-MS/MS, liquid chromatography–tandem mass spectrometry; LOD, limit of detection; LOQ, limit of quantification; RIA, radioimmunoassay.

**Table 2:** Assay and validation status of the porcine studies on which the matrix-specific conclusions of this review rest. Analytical criteria are appraised by platform: for immunoassays, parallelism, antibody cross-reactivity, recovery, limit of detection, and intra- and inter-assay coefficients of variation in the porcine matrix concerned; for chromatographic methods (HPLC with ultraviolet detection), separation selectivity, interference, calibration, extraction recovery, precision, and detection and quantification limits. ‘Not reported’ indicates that the information is absent from the source; ‘not assessable’ indicates that it could not be checked because the full text was unavailable, which is not evidence that the method lacked validation; immunoassay-specific criteria (parallelism, antibody cross-reactivity) do not apply to the chromatographic entries.

Matrix	Source	Assay and Platform	Analytical Validation Reported in Pigs	Biological Validation in Pigs
Saliva	Cook et al. [19]	In-house competitive ^3^H-cortisol RIA; rabbit antiserum against cortisol-3-oxime–BSA (Endocrine Sciences, Calabasas Hills, CA, USA); free fraction separated with dextran-coated charcoal. Whole saliva collected on a cotton swab chewed for less than 2 min, centrifuged, and stored at −80 °C	Sensitivity 0.55 nmol/L; intra-assay CV below 6% at 1–25 nmol/L; inter-assay CV 3.2–6.5% (quality controls prepared in assay buffer); antiserum cross-reactivity corticosterone 2.9%, desoxycortisol 4.5%, 21-desoxycortisol 6.8%, prednisolone 52%, prednisone 26%; agreement with a commercial solid-phase ^125^I RIA adapted for saliva, r = 0.81.	ACTH (200 IU i.v.) and 5 min nose-snare restraint in the same 6 pigs; serum–saliva correlation r = 0.88 (*p* < 0.025) and r = 0.80 (*p* < 0.05); salivary increment 164% and 236%. Road transport for 1 h (*n* = 10) raised salivary cortisol from 5.8 to 11.2 nmol/L (*p* < 0.0001)
Saliva	Ruis et al. [3]	Solid-phase RIA (Coat-A-Count Cortisol TKCO, Diagnostic Products Corp., Los Angeles, CA, USA)	Sensitivity 0.13 ng/mL; intra-assay CV 3.6–9.1%; inter-assay CV 7.4–9.8%; recovery 101–117%; linearity of dilution 106%; manufacturer cross-reactivity corticosterone 0.94%, cortisone 0.98%, 11-deoxycortisol 11.4%	Social isolation, 4 h; response present and time-of-day dependent (*p* < 0.05). No pharmacological challenge
Saliva	Wolf et al. [24]	Cortisol EIA, antibody raised against cortisol-3-CMO:BSA (Endocrine Research Laboratory, University of Pretoria, Pretoria, South Africa)	Sensitivity 0.6 ng/mL; intra-assay CV 9.5–11.0%; inter-assay CV 15.0–15.2%. Parallelism, recovery, and cross-reactivity not reported	ACTH challenge; median + 1100% against +400% in saline controls; descriptive statistics only
Saliva, urine, feces	Mohan et al. [100]	Single commercial cortisol ELISA (Labor Diagnostika Nord, Nordhorn, Germany) applied to all four matrices; no metabolite-specific antibody for feces	Not reported	None; resting animals, correlation study (plasma–saliva r = 0.77, *p* < 0.05)
Urine	Hay and Mormède [20]	Solid-phase extraction on C18 cartridges followed by HPLC with ultraviolet detection at 254 nm (method of [101]); creatinine by the Jaffe reaction; results expressed per µg creatinine	Intra- and inter-assay CV 7.4% and 10.6% for cortisol, 5.4% and 10.9% for cortisone. Other chromatographic criteria not reported; method described in [101]	Breed contrast rather than challenge; Meishan about five-fold above Large White (*p* < 0.0001). No plasma sampling
Urine	Hay and Mormède [101]	HPLC with ultraviolet detection—the analytical method paper for [20,21,22]	Not assessable (full text not obtained). The two studies applying the method report intra- and inter-assay CV of 7.4% and 10.6% for cortisol and 5.4% and 10.9% for cortisone [20,21]; selectivity, calibration, extraction recovery and detection limits are not restated there	Not applicable (analytical method paper)
Urine	Hay et al. [21]	Solid-phase extraction and HPLC with ultraviolet detection at 254 nm (method of [101]); plasma cortisol by immunoassay and ACTH by immunoradiometric assay validated for pig plasma; results expressed per µg creatinine	Urinary cortisol intra- and inter-assay CV 7.4% and 10.6%, cortisone 5.4% and 10.9%; plasma cortisol 7.6% and 12.5%; ACTH 3.0% and 7.8%. Creatinine correction validated against the measured excretion rate (cortisol r = 0.90, cortisone r = 0.92, *n* = 361, *p* < 0.0001). Other chromatographic criteria not restated (method of [101])	Dexamethasone suppression and ovine CRH stimulation; urinary cortisol fell and rose (both *p* < 0.0001); urinary peak 2–3 h
Urine	Pol et al. [22]	Solid-phase extraction and HPLC with ultraviolet detection at 254 nm (method of [101]); creatinine by dry-chemistry spectrophotometry (Ektachem DT II, Eastman Kodak Company, Rochester, NY, USA); expressed as cortisol-to-creatinine ratio in µg/g. Spontaneously voided urine, first spurt discarded, acidified with 6 M HCl and frozen	Not reported in the source; the method is referenced to [101]	Depot ACTH (1 mg i.m.) against saline, 10 sows each; urinary cortisol rose from 2 h, mean peak 3 h 40 min (range 2–5 h), basal again 9–24 h after injection; no circadian variation on the pre-injection day. Plasma not measured in the challenge
Feces	Möstl et al. [23]	11-oxoaetiocholanolone EIA, selected after screening HPLC fractions of infused 14C-cortisol with cortisol, corticosterone and 11-oxoaetiocholanolone EIAs	Not reported for porcine feces	ACTH and dexamethasone; inadequate in pigs—3 of 6 responded, no fecal suppression
Feces	Wolf et al. [24]	Group-specific EIA for 5α-pregnane-3β,11β,21-triol-20-one (5α-3β,11β-diol metabolites), Endocrine Research Laboratory, University of Pretoria	Sensitivity 1.5 ng/g dry weight; intra-assay CV 4.9–6.3%; inter-assay CV 9.5%. Parallelism, recovery and cross-reactivity not reported	ACTH challenge and road transport; +180% and +70%; descriptive statistics only
Feces	Asencio et al. [25]	Same group-specific 5α-3β,11β-diol EIA (Palme laboratory, University of Veterinary Medicine Vienna, Vienna, Austria).	Sensitivity 4 ng/g; intra-assay CV below 10%. Inter-assay CV, parallelism, recovery and cross-reactivity not reported.	Road transport, 3 h, and dexamethasone; *p* = 0.049; peak 12–24 h
Hair	Prims et al. [27]	Cortisol saliva ELISA (IBL International, Hamburg, Germany) validated for porcine saliva, applied to both saliva and hair; hair regrown over 21 days after shaving the dorsum at day 7, washed twice for 3 min in isopropanol, ground, extracted in methanol for 24 h	Intra-assay CV below 5% in both matrices; all samples run in a single assay; inter-assay CV unavailable. Parallelism, recovery, cross-reactivity and limit of detection not reported, and the assay was validated for porcine saliva rather than for porcine hair	Crowding with repeated mixing, 21 d; hair 87.3 against 75.6 pg/mg (*p* = 0.005); saliva not different
Hair	Heimbürge et al. [26]	Cortisol ELISA (Demeditec Diagnostics, Kiel, Germany); hair washed twice in isopropanol, methanol extraction; 1 cm segments in pigs; native and regrown hair both analyzed.	Sensitivity 1.1 pg/mg in pigs; intra-assay CV 4.5%;inter-assay CV 6.8%; parallelism confirmed (*p* > 0.05). Recovery and cross-reactivity not reported.	Repeated ACTH, 4 wk; negative in pigs (*p* = 0.10) and positive in cattle (*p* < 0.001) in the same experiment
Hair	Pollock et al. [28]	Cortisol ELISA (kit not reported) and DHEA ELISA (Salimetrics, Carlsbad, CA, USA); three 3 min methanol washes	For cortisol, only manufacturer cross-reactivity is given (prednisolone 0.568%, cortisone 0.130%); parallelism, recovery, limit of detection, and both CVs are reported for DHEA only	None; methodological study of wash protocol and contamination
Hair	Escribano et al. [30]	Cortisol and cortisone AlphaLISA (PerkinElmer, Waltham, MA, USA), previously validated for porcine hair; hair washed twice in isopropanol, methanol extraction	CV below 12%; limit of quantification 6.96 pg/mL for cortisol and 2.80 pg/mL for cortisone. Parallelism, recovery and cross-reactivity deferred to the earlier validation paper	Heat stress with against without cooling; hair cortisol unchanged, cortisone raised (*p* < 0.001)

ACTH, adrenocorticotropic hormone; BSA, bovine serum albumin; CMO, carboxymethyloxime; CRH, corticotropin-releasing hormone; CV, coefficient of variation; DHEA, dehydroepiandrosterone; EIA, enzyme immunoassay; ELISA, enzyme-linked immunosorbent assay; HPLC, high-performance liquid chromatography; i.m., intramuscular; IU, international units; i.v., intravenous; RIA, radioimmunoassay.

**Table 3:** Design features of the porcine studies supporting the principal recommendations of this review. Entries are restricted to information extracted for this review; ‘not extracted’ marks features that were not recorded during extraction and should not be read as absent from the source.

Study (Matrix)	Design and Comparison	Experimental Unit and *n*	Repeated Measurements	Principal Design Limitations
Cook et al. [19] (saliva)	ACTH (200 IU i.v.) and 5 min nose-snare restraint with matched serum and saliva; 1 h road transport against an own-control day	Individually penned female pig, about 100 kg; *n* = 6 (ACTH, then snare in the same pigs); *n* = 10 (transport)	Matched samples every 20 min for 3 h (ACTH) and every 5 min for 30 min (snare); 5 control-day and 6 transport-day saliva samples per pig	No saline control for ACTH; both challenges applied sequentially to the same 6 catheterized pigs; the saliva-to-serum ratio varied with concentration (6.1–16.4% under ACTH)
Ruis et al. [3] (saliva)	Social isolation for 4 h at 12 and 20 weeks of age; circadian profiling	Individual pig; *n* not extracted	Repeated saliva sampling across the day	No pharmacological challenge; the response depended on the time of day at which isolation began, so timing modifies the effect estimate
Wolf et al. [24] (saliva, feces)	ACTH analog against saline; road transport	Individual gilt; 4 ACTH-treated, 2 saline	Serial sampling; descriptive statistics only	Very small groups; saline controls also rose (+400%), indicating a handling effect; no inferential test
Hay and Mormède [20] (urine)	Breed contrast (Meishan against Large White); no challenge	Individual sow; *n* not extracted	Not extracted	Breed differs in more than adrenocortical activity; no plasma sampling
Hay et al. [21] (urine)	Within-animal dexamethasone suppression and CRH stimulation	Catheterized pregnant sow; *n* = 10	Continuous urine collection; within-animal comparison	Catheterized animals are not representative of field sampling; untreated control group not extracted
Pol et al. [22] (urine)	Depot ACTH against saline (experiment 1); individual against group pens during gestation (experiment 2)	Tethered multiparous pregnant sow; 10 ACTH-treated, 10 saline. Housing: 96 gilts in 4 batches, cortisol analyzed for 2 batches; unit = sow in individual pens and pen of 6 in group pens (4 pens)	Spontaneously voided urine collected over 3 consecutive days and pooled per 3 h; housing: before and 4 h after transfer on days −6 and 108 of gestation	Plasma not measured in the challenge, so the urinary delay cannot be referenced to plasma; ACTH data pooled across gestation weeks 6, 9 and 12; only 4 group pens analyzed for cortisol in the housing comparison, which limits power
Möstl et al. [23] (feces)	ACTH and dexamethasone challenge	Individual pig; *n* = 6	Serial fecal sampling	Very small group; 3 of 6 animals responded; assay not matched to porcine metabolites
Asencio et al. [25] (feces)	3 h road transport; dexamethasone	Fattening pig; *n* = 36	Treatment × time analysis	Transport combines handling, loading, and the journey, so the response cannot be attributed to a single component
Prims et al. [27] (hair, saliva)	Crowding with repeated mixing for 21 d	Artificially reared piglet; *n* = 24	Hair regrown over 21 d; saliva sampled	Artificial rearing limits generalization; pen-level effects require consideration in group-housed designs
Heimbürge et al. [26] (hair)	Repeated ACTH over 4 weeks against untreated controls; cattle in parallel	Gilt; *n* = 38	Native, regrown, and segmented hair	In hair segments, untreated controls exceeded treated animals (*p* < 0.05), indicating unexplained between-group differences
Escribano et al. [30] (hair)	Heat stress in uncooled against cooled rooms	Treatment applied by room; numbers not extracted	Not extracted	Treatment is confounded with room, so room-level replication determines the strength of inference
Six-farm survey [35] (serum, oral fluid)	Observational comparison with structured welfare scores	Farm; 6 farms; 29 serum and 30 oral fluid samples	None	Few farms; observational design without control of farm-level confounders

ACTH, adrenocorticotropic hormone; CRH, corticotropin-releasing hormone; IU, international units; i.v., intravenous.

**Table 4:** Cortisol concentrations, response magnitudes, and related endocrine findings reported in pigs, by matrix and condition. Values were obtained with differing assays and normalization practices; urinary concentrations in ng/mL are not creatinine-corrected. Relative changes describe the response within the source study only and are not comparable across studies or matrices.

Animal Category	Matrix	Condition	Reported Value	Relative Change Within the Source Study	Source
Pigs, all categories	Serum	Literature benchmark	27.5–31.8 ng/mL	not reported	[34]
Pigs, commercial farms	Serum/oral fluid	Survey against that benchmark	Most values outside the interval	not reported	[35]
Pigs	Saliva	Fraction of serum concentration	8.6–13.3%	not reported	[19]
Pigs, barrows in metabolic cages	Urine, uncorrected	12-h collections	0.6–94.3 ng/mL; most values <20 ng/mL	not reported	[109]
Sows, stable group	Urine, uncorrected	Baseline	≈8 ng/mL	not reported	[10]
Sows, recently mixed	Urine, uncorrected	Social challenge	≈30 ng/mL	about +275% vs. stable group	[10]
Sows, weaning	Urine, normalization not extracted	24–48 h post-weaning	3–4-fold increase	+200 to +300%	[12]
Sows, feed deprivation	Urine, normalization not extracted	12 h/24 h	+35–40%/+80–120%	+35 to +40%/+80 to +120%	[11]
Female pigs	Saliva	ACTH challenge	≈+1100%	about 12-fold	[24]
Female pigs	Feces	ACTH challenge	≈+180%	about 2.8-fold	[24]
Female pigs	Feces	Transport	≈+70%	about 1.7-fold	[24]
Fattening pigs	Feces	3 h road transport	Peak 12–24 h; baseline by 24–48 h	not reported	[25]
Sows	Hair	Farrowing crate vs. loose housing	No significant difference	No significant change	[31]
Growing pigs	Hair	High ambient temperature	Cortisone/cortisol ratio informative	not reported	[30]
Finishing pigs	Hair	20 commercial farms	Variation within batch exceeds variation between farms	not reported	[32]
Pigs, mixed organic farms	Hair	Season, system, sex	All three affect the concentration	not reported	[33]
Sows and piglets	Hair	Shortened lactational restraint	Concentration decreased in both	not reported	[116]
Cattle and pigs	Hair	Repeated ACTH administration	Increased in cattle; no increase in pigs	Species-dependent response	[26]
Piglets	Hair and saliva	Chronic stress model	Hair higher under crowding/mixing; saliva not different	Hair: 87.3 vs. 75.6 pg/mg; *p* = 0.005	[27]
Swine hair	Hair	Wash protocol and contamination	Both alter measured cortisol and DHEA	not reported	[28]
Pigs	Plasma/saliva	Weaning	HPT and HPA axes both altered	not reported	[75]
Sows	Serum	Postpartum dysgalactia syndrome	Differs from clinically healthy sows	not reported	[86]
Periparturient sows	Plasma	Thermal environment	HPA hormones and oxytocin altered	not reported	[61]
Primiparous sows	Plasma	Controlled heat stress, late gestation	Thermoregulation and metabolite profile affected	not reported	[62]
Sows	Plasma	Oral cortisol during gestation	Offspring birth weight and ACTH response reduced	not reported	[81]

–, not reported. ACTH, adrenocorticotropic hormone; DHEA, dehydroepiandrosterone; HPA, hypothalamic–pituitary–adrenal; HPT, hypothalamic–pituitary–thyroid.

**Table 5:** Degree of invasiveness together with the practical and analytical specifications governing the choice between the four matrices in pigs.

Property	Saliva	Urine	Feces	Hair
Degree of invasiveness	Non-invasive rope collection; minimally invasive if individual restraint is required	Non-invasive; no contact	Non-invasive; no contact	Minimally invasive; brief restraint and clipping
Analyte measured	Free cortisol and cortisone	Cortisol plus glucuronide and sulfate conjugates	Glucocorticoid metabolites after hepatic and intestinal transformation	Cortisol and cortisone during growth
Period integrated by a sample	Minutes	Interval since the previous void	Period of gut passage; varies with diet and age	Potentially weeks to months; not established in pigs
Delay after an event	Minutes	Peak 2–5 h after ACTH; not a measured plasma lag [22]	12–24 h to peak [25]	Not established in pigs
Duration of detectable response	Baseline within 5–24 h after ACTH [24]	Baseline 9–24 h after depot ACTH [22]	Baseline within 24–48 h after transport [25]	Not established in pigs
Relation to plasma	8.6–13.3% of serum [19]	No plasma correlation established in [20,21]; normalization validated against excretion rate [21]	Not expressible as a fraction	Not expressible as a fraction
Assay species-specificity	Low; cortisol is the analyte [96]	Moderate; conjugation patterns differ	High; requires a 5α-3β,11β-diol assay [23,24]	Low, but extraction and wash steps are decisive [28]
Normalization required	None established	Creatinine [20]	Per g dry or wet feces [93]	Per mg hair; segment length defines the window
Principal preanalytical risk	Device-dependent recovery	Freeze–thaw cycles	Post-defecation bacterial metabolism [23,24]	External contamination and wash protocol [28]
Group-level sampling	Feasible with rope sampling [35]	Not feasible; individual voids	Pen-level sampling feasible; individual attribution requires identification	Not feasible; individual clipping
Response to controlled challenge	Consistently detected [24]	Consistently detected [20,22]	Detected with a matched assay [24,25]	Not consistently detected [26,27,95]
Reference intervals in pigs	Not established	Not established	Not established	Not established

ACTH, adrenocorticotropic hormone.

## Data Availability

No new data were created or analyzed in this study. Data sharing is not applicable to this article.

## Abbreviations

The following abbreviations are used in this manuscript:

11β-HSD2	11β-hydroxysteroid dehydrogenase type II
ACTH	Adrenocorticotropic hormone
BSA	Bovine serum albumin
CLIA	Chemiluminescent immunoassay
CMO	Carboxymethyloxime
CRH	Corticotropin-releasing hormone
CV	Coefficient of variation
DHEA	Dehydroepiandrosterone
EIA	Enzyme immunoassay
ELISA	Enzyme-linked immunosorbent assay
HPA	Hypothalamic–pituitary–adrenal (axis)
HPLC	High-performance liquid chromatography
HPT	Hypothalamic–pituitary–thyroid (axis)
IU	International units
LC-MS/MS	Liquid chromatography–tandem mass spectrometry
LOD	Limit of detection
LOQ	Limit of quantification
PLF	Precision livestock farming
RIA	Radioimmunoassay
